# Electrophysiology Reveals That Intuitive Physics Guides Visual Tracking and Working Memory

**DOI:** 10.1162/opmi_a_00174

**Published:** 2024-11-22

**Authors:** Halely Balaban, Kevin A. Smith, Joshua B. Tenenbaum, Tomer D. Ullman

**Affiliations:** Department of Brain and Cognitive Sciences, Massachusetts Institute of Technology, Cambridge, MA, USA; Department of Psychology, Harvard University, Cambridge, MA, USA; Department of Education and Psychology, The Open University of Israel, Ra’anana, Israel

**Keywords:** intuitive physics, object tracking, working memory, pointer system, violation of expectation

## Abstract

Starting in early infancy, our perception and predictions are rooted in strong expectations about the behavior of everyday objects. These intuitive physics expectations have been demonstrated in numerous behavioral experiments, showing that even pre-verbal infants are surprised when something impossible happens (e.g., when objects magically appear or disappear). However, it remains unclear whether and how physical expectations shape different aspects of moment-by-moment online visual scene processing, unrelated to explicit physical reasoning. In two EEG experiments, people watched short videos like those used in behavioral studies with adults and infants, and more recently in AI benchmarks. Objects moved on a stage, and were briefly hidden behind an occluder, with the scene either unfolding as expected, or violating object permanence (adding or removing an object). We measured the contralateral delay activity, an electrophysiological marker of online processing, to examine participants’ working memory (WM) representations, as well as their ability to continuously track the objects in the scene. We found that both types of object permanence violations disrupted tracking, even though violations involved perceptually non-salient events (magical vanishing) or new objects that weren’t previously tracked (magical creation). Physical violations caused WM to reset, i.e., to discard the original scene representation before it could recover and represent the updated number of items. Providing a physical explanation for the violations (a hole behind the occluder) restored object tracking, and we found evidence that WM continued to represent items that disappeared ‘down the hole’. Our results show how intuitive physical expectations shape online representations, and form the basis of dynamic object tracking.

## INTRODUCTION

As they go about in the world, people have strong expectations about the physical behavior of everyday objects. Objects do not simply disappear and reappear, and solid bodies do not pass through one another or break apart for no reason. Such expectations, which are at the heart of our ‘intuitive physics’, help us to efficiently perceive, predict, and interact with our surroundings (Kubricht et al., 2017; Spelke, 1990). However, it remains largely unknown whether and how such physical expectations directly affect active visual processing in terms of object tracking and scene representation. Here, we sought to answer these questions, via novel use of EEG markers that reveal the unfolding dynamics in the mental processing of physical expectations.

The fundamental role intuitive physics plays in the ability to make sense of the world can be clearly seen in developmental and comparative findings. Numerous studies with human infants and non-human animals have reported behavioral markers of surprise (e.g., differential looking times) when observers are presented with events that violate physical principles such as cohesion or continuity, as compared with highly similar events that control for different non-physical factors (e.g., Baillargeon et al., 1985; Cacchione & Krist, 2004; Wynn, 1992; Xu & Carey, 1996). This rich literature collectively suggests that there is a small set of non-verbal core physical expectations that is in place by the first year of people’s lives, and shared by other animals (Spelke & Kinzler, 2007).

On the basis of core physical reasoning, people continue to acquire and refine their intuitive physical knowledge from childhood to adulthood. While the specific format of this physical reasoning and its development remain an active topic of debate (e.g., Battaglia et al., 2013; Fischer et al., 2016; Gilden & Proffitt, 1989; Lerer et al., 2016; Piloto et al., 2022; Proffitt et al., 1990; Ullman et al., 2017), here we focus on a relatively format-neutral question: How do basic physical expectations shape the online processing of objects, when people are not explicitly required to perform physical judgements or inferences?

Several studies have used traditional cognitive paradigms to test the effects of intuitive physics on online visual processing, producing a mixed pattern of results. One study relied on a modified object-review paradigm (Kahneman et al., 1992) where participants saw two letters inside placeholders in a preview display, after which the letters disappeared and the placeholders alone moved in different ways, and finally a single letter appeared in one of the placeholders. Participants had to indicate, as quickly as possible, whether this letter previously appeared in the preview display (in any of the placeholders). The classic finding in this paradigm is that when a letter repeats, responses are faster when it reappears inside the same placeholder as in the preview display than in a different placeholder. This effect is referred to as an object-specific preview benefit, and interpreted as reflecting the dynamics of object file updating. Mitroff and colleagues compared a placeholder splitting (resulting in three placeholders at test) during the movement to a condition with only a change in trajectory (i.e., the placeholder following the trajectory of one of the post-split placeholders) as well as to a condition with straight and unchanged motion paths, and found that the effect persists following these violations of cohesion, and it is even larger than for objects that remain intact, but is smaller than in trajectory-change trials (Mitroff et al., 2004). This suggests that object files are not completely independent from physical principles, nor are they fully dependent on them.

Related findings were also obtained in the multiple object tracking paradigm (MOT; Pylyshyn & Storm, 1988). MOT usually involves presenting several identical items, briefly marking a subset of them as targets, and then asking participants to track these targets as all items (now again identical) move for several seconds. Tracking performance is quantified as accuracy in identifying all targets when items stop moving, or in reporting whether a cued item was a target or not. Using this paradigm, it has been shown that people are better at tracking targets that move like rigid objects than when they have substance-like motion properties (vanMarle & Scholl, 2003). However, this result was not due to violations of cohesion or rigidity, but specifically due to the repeated extension and shrinkage involved in substance-like movement. This means that the effect might reflect spatial ambiguity, and not physical expectations per se. Aside from physical expectations, the role of physical prediction in object tracking is also somewhat unclear: on the one hand, it is well established that under most conditions in MOT tasks people do not spontaneously predict the future trajectories of object by extrapolation (Franconeri et al., 2012; Keane & Pylyshyn, 2006), but on the other hand it has been shown that objects that move according to physical principles are easier to track (Lau & Brady, 2020).

In short, while core physical knowledge is fundamental and is a likely basis for much of intuitive physical reasoning, its involvement in object tracking and visual scene processing is still not well understood. Here, we sought to use a novel EEG paradigm to examine whether task-irrelevant physical expectations will affect the online processing of objects. We specifically targeted working memory (WM), the mental workspace that holds information in an active state, ready to be accessed and manipulated (Baddeley, 1992; Luck & Vogel, 1997). Visual WM is involved not only in classic memory paradigms, but whenever material has to be held online, such as in search or imagery contexts (Carlisle et al., 2011; Hyun & Luck, 2007; Luria & Vogel, 2011b; Tsubomi et al., 2013).

Our specific goal was to study whether and how physical expectations affect two distinct aspects of online object tracking and scene processing in working memory. The first aspect is the *content* of visual WM. In particular, we asked whether representations in WM reflect the high-level physical interpretation of a scene, even if it contradicts low-level visual information or task demands. The second aspect is the ongoing *correspondence* between WM content and perception, implemented by a ‘pointer system’ (Kahneman et al., 1992; Pylyshyn, 2000, 2001). A pointer is a one-to-one index that maps a representation in visual WM to a specific part of perception, allowing it to be accessed and updated when the analogous real-world object changes. For example, when a peacock spreads its tail, your visual impression changes dramatically, but you still consider it the same peacock. The sense in which these different visual impressions are still the same object is supported by a continuous pointer that connects them to the same WM representation. Importantly, WM representations and pointers are both theoretically and empirically distinct (Balaban et al., 2023; Kahneman et al., 1992; Pylyshyn, 2000, 2004), with pointers serving as the content-free individuation infrastructure for the information that is stored in WM.

If something disrupts the ongoing correspondence between a WM representation and perception (for example, a single object inexplicably splits into two objects), WM can no longer track and update its original object representations. Without a valid mapping, WM has been found to ‘reset’, meaning it removes unmapped representations, and creates new representations and mappings (Balaban et al., 2018a, 2019; Balaban & Luria, 2017). This resetting process has distinct neural and behavioral characteristics, and is accompanied by an inability to use the unmapped representations to track objects (Balaban et al., 2018b). With this background in mind, our second question was whether violations of physical expectations disrupt the pointer system’s ability to track objects, which would suggest that visual object tracking depends on high-level physical expectations.

**Figure 1.**
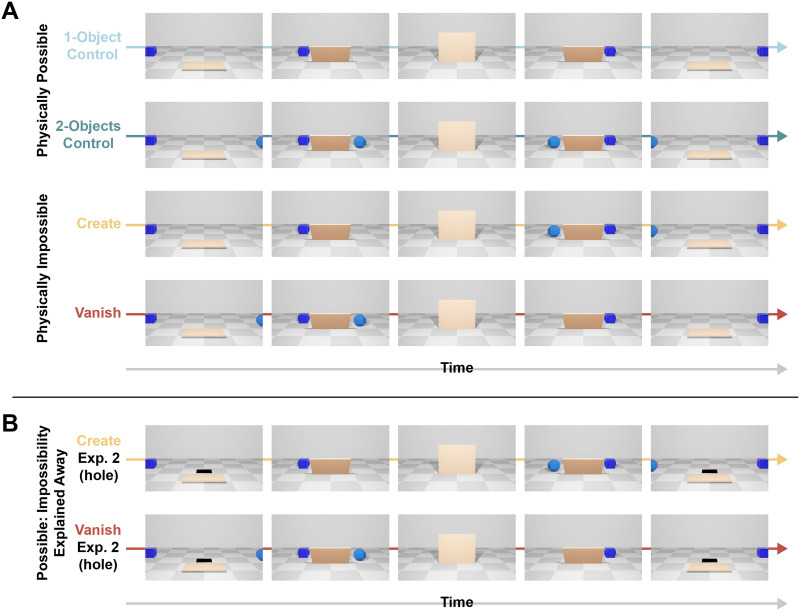
Frames from the animations used as stimuli. (A) Conditions of Experiment 1. Top to bottom: 1-Object and 2-Objects Controls, Create, and Vanish. Note that Create starts like 1-Object and ends like 2-Objects, while Vanish is the opposite. (B) The Create and Vanish conditions of Experiment 2. Note that during the most critical part of the trial (when items exit the occluder), these conditions are identical to the corresponding conditions in Experiment 1.

We modified a standard WM paradigm, to include stimuli based on classic developmental studies (Wynn, 1992) and recent AI-benchmarks probing physical expectations in machines (Piloto et al., 2022). In two experiments, people watched short animations of one or two objects crossing a stage (Figure 1). The scenes either unfolded as expected (Videos S1 and S2), or contradicted object permanence by having an object appear or disappear behind the occluder (i.e., magical creation or vanishing; Videos S3 and S4). While behavioral performance in such tasks is potentially useful and important, it is known that task effects in online processing may not reflect WM or object tracking, but rather sub-stages that precede or follow them, such as perception or decision making (Awh et al., 2007). Instead, we focused on electrophysiological markers that are specific to different aspects of online processing in WM. Using EEG, we recorded scalp electrical activity as people watched the animations in Figure 1, and tested for the first time how violations of physical expectations are dynamically processed in online object tracking and representation.

To evaluate moment-by-moment active processing, we isolated contralateral delay activity (CDA; see McCollough et al., 2007; Vogel et al., 2005; Vogel & Machizawa, 2004), an event-related potential (ERP) index of visual WM. The excellent temporal resolution of ERPs allowed us to examine different predefined time-windows of the CDA amplitude, to uncover evolving mental processing in a fine-grained manner.

**Figure 2.**
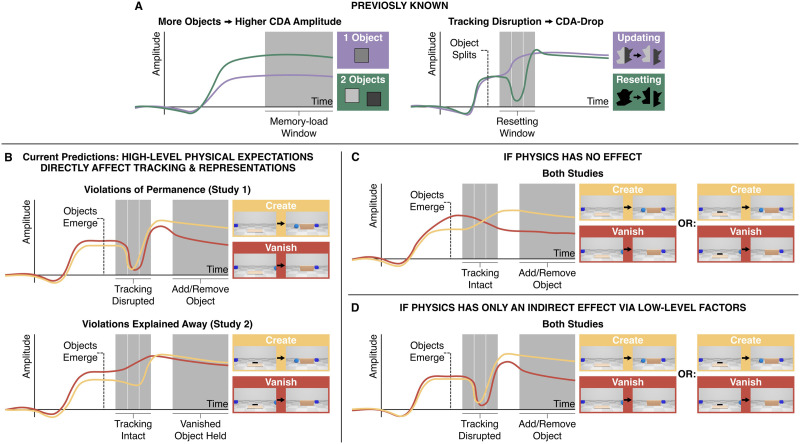
Schematic of past results and predictions for the current experiments. (A) Previous CDA-based findings. Left: the CDA amplitude is higher when more objects are maintained in working memory (e.g., Vogel & Machizawa, 2004). Right: If pointer system tracking is disrupted (e.g., when an object splits in two), the CDA amplitude drops, while events that allow uninterrupted tracking (here, because each half is associated with a different color already before they split) involve a steady change in amplitude (e.g., Balaban & Luria, 2017). (B) Current predictions, based on the hypothesis that intuitive physics expectations guide tracking and representations. In Experiment 1 (top), violations of object permanence should disrupt tracking and trigger a resetting CDA-drop, followed by the addition or removal of items from working memory. In Experiment 2 (bottom), visually-identical events should not disrupt tracking when the violations are explained away by the presence of a hole (there might still be a residual effect in the Create condition, given that objects emerging from a hole go against gravity), and the new construal of events should lead the removed object to remain in working memory. (C) Alternative predictions based on the hypothesis that high-level physical expectations have no effect over and above perceptual factors. Experiments 1 and 2 should have identical patterns, because they are identical aside from the physical explanations and expectations involved. Specifically, no condition should include a CDA-drop, because none of the events include a salient change to a tracked object, and the representations should smoothly transition to add or remove object. (D) Alternative predictions based on the hypothesis that physics has only an indirect effect, through low-level visual cues and kinematics. Again, Experiments 1 and 2 should have identical patterns, but here the prediction is that both include a drop before an object is added or removed.

Two different ways of analyzing the CDA tap into the distinct aspects of online processing targeted in the present work, namely the *content* of WM, and the *correspondence* between WM representations and perception, via the pointer system. To the question of content: the CDA’s amplitude rises when more items are held in online processing (Figure 2A, left). This rise is correlated with an individual’s WM capacity, and immune to related factors such as task difficulty or spatial attention (for a review, see Luria et al., 2016). Unlike behavioral measures, the CDA factors out contributions of perceptual facets (Ikkai et al., 2010; Luria et al., 2010; Ye et al., 2014), by using a bilateral paradigm. It also factors out response-related facets, by focusing the analysis on the period preceding the test phase, meaning before participants can initiate any response. Comparing the CDA amplitude across conditions indicates how much information is represented in WM (e.g., Adam et al., 2018; Peterson et al., 2015; Wang et al., 2019). Here, we applied the same logic to whole scenes, for the first time, to establish whether and how WM representations are adjusted following physically impossible events.

As mentioned, beyond the content of WM, the CDA can also be used to decipher pointer system dynamics: the resetting process that is triggered by invalidating the WM-perception correspondence (e.g., if an object splits in two) is accompanied by a reliable transient drop in CDA amplitude (Figure 2A, right; Balaban et al., 2018a, 2019; Balaban & Luria, 2017). The CDA-drop specifically indicates pointer system disruption, and does not reflect related factors such as general surprise (see Balaban & Luria, 2019, and also the Discussion). We tested whether physically impossible events are associated with a CDA-drop, which would suggest that these violations disrupt the pointer system’s ability to track objects in the scene.

We hypothesized that task-irrelevant physical expectations shape online processing, both in terms of how representations in WM are modified to reflect dynamic scene information, and in terms of the ability to continuously track the objects in the scene. In Experiment 1, we expected violations of object permanence to interrupt object tracking, causing a CDA-drop in the previously-established resetting window (*correspondence*), followed by the appropriate removal or addition of an object from WM (*content*; see Figure 2B, top). In Experiment 2, we added a hole to the scene (Videos S5 and S6). This potentially makes the creation or vanishing of an object no longer impossible, but importantly keeps the dynamics of objects and perceptual portions of the scene exactly the same as Experiment 1. Because violations could now be explained away, we expected the modified expectations about physical dynamics to minimize tracking disruption, especially for the Vanish condition, leading to no resetting, and also no removal of the vanished object from WM (Figure 2B, bottom).

There are several alternative patterns of results that are a-priori possible given the setup above. In particular, current theories of the pointer system depict it as a relatively low-level system that relies on visual and spatiotemporal information (for a recent review, see Holcombe, 2023). So, one attractive alternative hypothesis is that physical expectations do not guide object tracking or WM representations (Figure 2C). If so, we should expect to find the same pattern of results in both experiments. More specifically, in both experiments there should be no resetting effect in any condition, given that the violations do not involve any salient visual change (Vanish) or involve aspects of the scene which were not previously tracked (Create). Additionally, in both experiments we should see the same addition and removal of objects, based only on the visible dynamics of the scene and not the underlying physical explanation. A different hypothesis is that intuitive physics does affect the pointer system and the representations in WM, but only indirectly through low-level cues and basic influence on the kinematics of the situation (Figure 2D). If this is true, we should again expect to find the same pattern of results in both experiments, but this time the hypothesized pattern should always include resetting, and the addition or removal of objects (as the high-level explanation provided by the hole cannot undo the visual impression of creation and vanishing).

## METHODS

### Participants

Participants were students with normal or corrected-to-normal visual acuity and normal color-vision, who gave informed consent following the procedures of a protocol approved by the Massachusetts Institute of Technology Committee on the Use of Humans as Experimental Subjects under protocol 1912000059. Due to the COVID-19 global pandemic, the experiments were run at Tel Aviv University, Israel. Participants were notified of their rights before the experiment, were free to terminate participation at any time, and were compensated monetarily for their time at a rate of $30 an hour.

Each experiment included 16 naïve participants (11 females, mean age 24 in Experiment 1, and 14 females, mean age 24 in Experiment 2). Sample size was determined based on the smallest effect size in a previous study with the most similar analysis method (Balaban & Luria, 2017), which was *d* = 0.8, entailing 16 participants for 80% power. Note that this power analysis did not target across-experiments comparisons, which we now report as post-hoc analyses. Additionally, our power calculations did not take into account the possibility that the current setup will be less temporally-precise than previous studies, due to the nature of the events used as stimuli, which could introduce more variability. Because the experiment required holding fixation and avoiding blinks for a relatively long time (roughly 6 seconds on each trial), participants who could not perform the task under these limitations were released after the first few blocks and replaced (3 in each experiment). Another participant in Experiment 1 was released and replaced for failing to understand the task, and another one was released and replaced due to electrode malfunction.

### Stimuli and Procedure

**Figure 3.**
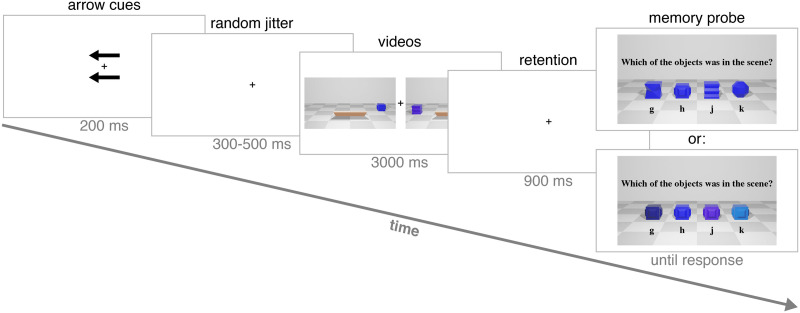
Example of a trial sequence in Experiments 1 and 2. Black arrows indicated to participants which side is relevant for the upcoming trial. The video animations included objects crossing the floor, as an occluder went up and down, briefly hiding them from view. Objects’ movement and the occluder were irrelevant to the task. After a retention interval, subjects selected the previously presented object from 4 options, which were either all the same color and varying in shape (a shape test; top), or all the same shape and varying in color (a color test; bottom).

In this study, we presented videos that may or may not contain a physical violation, and we measured EEG to examine how the scenes are represented. As is standard in CDA studies, this requires showing two videos—one for each hemisphere—and pre-cuing which side to pay attention to. Using this lateralized display allows defining the contralateral and ipsilateral hemispheres, and subtracting them to get rid of any common perceptual factors. To ensure participants pay attention to the videos and hold objects in WM, after the animations’ presentation they were given a task, to pick out one of the objects that they had seen from a set of objects. Figure 3 presents the trial sequence.

Each trial started with a 1,000 ms display of a fixation black cross (0.7° × 0.7° of visual angle from a viewing distance of approximately 60 cm) in the center of a grey screen (RGB values: 180, 180, 180). Then, two black arrows (3° × 1°) appeared above and below fixation for 200 ms. The arrows pointed either left or right randomly (with an equal probability), and this indicated the side to which participants were asked to attend for the upcoming trial. After another 300–500 ms (randomly jittered) fixation display, a video animation was played on each side of the fixation. Each video spanned about 21° in width and 12° in height, and was placed at a distance of 4.2° from the fixation, and at the middle of the screen’s height. The videos on both sides were always from the same condition (see below; from here on, when describing the number of items, we always refer to the relevant side), with all other details drawn randomly (without replacement) independently between the sides.

Animations were created in Blender, and rendered in the Eavee engine at 24 frames per second. Videos displayed a light-grey and white checkerboard pattern floor, a white back wall, and a rectangular light-orange screen, referred to as the occluder, in the middle of the scene. The occluder started off on the floor, went up until it stood vertically midway through the video, and lowered back down. Additionally, each video included one or two objects crossing the floor from one side to the other, with the occluder hiding them from view for 625 ms. When the animation included two objects, they moved in opposite directions, and one of them was slightly closer to the occluder than the other, so that they would not collide.

There were 4 possible un-nameable shapes, and 4 possible colors that were all shades of blue (the exact rendered color changed across the object’s surface because of the irregular shapes’ shading; RGB values are reported based on one representative area: 45, 45, 142; 60, 60, 230; 105, 60, 220; 45, 130, 210), for a total of 16 possible objects. Each object’s movement direction (from right to left or from left to right), color, and shape were determined randomly without replacement in each trial.

On the main trials, the videos played for 3 seconds. On catch trials, the videos played for only 300 ms. The goal of these short trials was to ensure participants paid attention not only to the end of the animation. They made up 25% of the trials in the first block, and 10% in all other blocks. Catch trials were not analyzed as part of our main examination of behavioral performance, but see the Supplemental for the full results. Importantly, we did not use catch trial performance (or, indeed, any measure of behavioral performance) to exclude participants, because this has the potential to bias the results in different ways. We note that this choice to include all participants who complied with the task has the potential of introducing more noise overall.

After the video animations, a 900 ms retention interval was presented, with only the fixation visible on screen. Then a memory probe appeared, including an image of 4 objects, from which participants chose the one that appeared in the video they saw on the relevant side of the screen. In conditions with a single object at the end of the trial (the 1-Object Control and Vanish conditions), that object was always presented during the test, along with three other objects that varied either in color or shape. In conditions with two objects at the end of the trial (the 2-Objects Control and Create conditions), the probed object was randomly selected from among these two. In color-test trials, the probed object’s shape was presented during the test, in all four possible colors. In shape-test trials, all four shapes were presented during the test, all in the probed object’s color. This means that in trials with two items, one of the unprobed object’s features (either its color or its shape) was shown at test, but not the presented object in its integrated form. Participants’ task was always to choose the combination of color and shape that appeared during the animation. Responses were made via button press (using the “g”, “h”, “j”, and “k” keys on a standard keyboard, based on the objects’ location in the image, from left to right). Responses were unspeeded and no feedback was provided. Participants were asked to hold fixation throughout the trial, and to blink only when they press the response key.

Participants completed 15 practice trials, followed by 16 experimental blocks of 50 trials each, for a total of 800 trials (including catch trials), and about 180 trials per condition (in line with the number of trials per condition used in previous studies, e.g., Drew & Vogel, 2008; Feldmann-Wüstefeld et al., 2018; Luria & Vogel, 2011b; Tsubomi et al., 2013). The experimental session took 2.5–3 hours (including EEG preparation).

There were 4 conditions, varying in the number of objects at the beginning and end of the animation (see Figure 1A). In the two physically possible Control conditions, either one or two objects simply passed behind the occluder (the 1-Object and 2-Objects Controls, respectively). There were two physically impossible conditions: Create, where one object went behind the occluder but two exited, and Vanish, where two objects went behind the occluder but only one exited. Thus, in the Create condition the video’s first half was identical to the 1-Object Control and its second half was identical to the 2-Objects Control, and vice versa for the Vanish condition. The Create and Vanish conditions constitute violations of object permanence, which translates to a change in the number of objects, allowing us to leverage the CDA’s set-size sensitivity (see below). In the Vanish condition, the item that disappeared was never probed, though in Experiment 1 participants were not explicitly told that, to allow them to interpret the scenes naturally.

There were only two differences between Experiments 1 and 2. First, in the Create and Vanish conditions of Experiment 2, a small black rectangle was placed right behind the occluder throughout the trial (see Figure 1B). Importantly, this black area was only visible when the occluder was down (i.e., at the beginning and end of the trial). The second difference compared to Experiment 1 was the explanation provided to participants before the experiment started. Participants in Experiment 2 were told that the black area is a hole in the floor, meaning that if the trial starts with two objects, one is going to fall down, and if the trial starts with one object, one is going to “climb up” from the hole, using a hidden leverage. In the Vanish condition, the location of the hole made it clear which object will disappear (always the front one), which participants were also told. They were further told that in the Vanish condition they can tell in advance which object is going to fall down the hole and disappear, due to the spatial layout of the scene. Participants were also shown example videos of the different conditions, and a demonstration of what the Vanish condition would look like if the occluder wasn’t there (showing an object falling down the “hole”).

### EEG Recording and Analyses

EEG was recorded inside a shielded Faraday cage, using a BioSemi ActiveTwo system, from 32 scalp electrodes placed at a subset of the extended 10–20 system’s locations, and from two electrodes placed on the mastoids, which served as reference. EOG was recorded from two electrodes placed 1 cm from the external canthi, and from an electrode placed 2 cm beneath the left eye. Data was digitized at 256 Hz.

Offline signal processing was performed using the EEGLAB (Delorme & Makeig, 2004) and ERPLAB (Lopez-Calderon & Luck, 2014) toolboxes, and custom Matlab (The Mathworks, Inc.) scripts. All electrodes were referenced to the average of the left and right mastoid electrodes. Continuous data was segmented into epochs from −200 to +3900 ms from animations’ onset (corresponding to the end of the retention interval). To remove blinks and eye-movements, artifact detection was performed on the EOG electrodes using a sliding window peak-to-peak analysis, with a threshold of 80 *μ*V. This procedure resulted in a mean rejection rate of 9.2% in Experiment 1, and 9.5% in Experiment 2. Rejection rates were comparable in all conditions, ranging 8-11%, without a statistical difference between them, and the residual eye-movements were overall very small and did not differ across conditions (for evidence that eye movements are not responsible for the CDA or the resetting-drop, see Balaban et al., 2018a; Balaban & Luria, 2017; Kang & Woodman, 2014, and and for an analysis of the HEOG across conditions, see the Supplemental). For plotting purposes, the epoched data were low-pass filtered using a noncausal Butterworth filter (12 dB/oct) with a half-amplitude cutoff point at 30 Hz. Statistical analysis was performed on the unfiltered data, to avoid potential effects of filtering on the results.

Epoched data were averaged separately for each condition, and difference waves were calculated by subtracting ipsilateral from contralateral activity, relative to the memorized side on each trial. To increase power, we did not exclude incorrect trials from the analysis, because in the present task (which was selected only to make sure WM encodes the items in the videos), errors do not necessarily correspond to trials where items are absent from WM during the critical time-period, given that the test involved fine-grain feature differences, and occurred a long time after some of the critical time-windows. Analyzing only trials with a correct response resulted in the same pattern of results, though some of the effects were weaker due to increased noise.

As is common in similar CDA studies (e.g., Balaban & Luria, 2017), we focus on the results from the average of 3 parietal-occipital electrode pairs—P7/8, Po3/4, and Po7/8—but similar patterns of activity were found in each pair separately.

In order to examine the maintenance and tracking of online representations in WM during a physics-violation task, we isolated the CDA (Luria et al., 2016; Vogel & Machizawa, 2004; Vogel et al., 2005). The CDA is an ERP component reflecting online processing in visual WM. The CDA amplitude rises with the number of items that are maintained in WM at each moment (and not with the number of items presented, or the number of active locations), in a manner that is tightly linked to an individual’s behavioral WM capacity (Luria et al., 2016). It was first reported in memory tasks, but can be measured equally well when items are held in WM while being completely visible (Tsubomi et al., 2013), like in search or tracking tasks (Drew & Vogel, 2008; Luria & Vogel, 2011b). Furthermore, CDA amplitude changes throughout a trial following the dynamics of WM-load, for example rising when items are added, or decreasing when items are chunked together (e.g., Luria & Vogel, 2014; Vogel et al., 2005). Numerous studies have shown that CDA amplitude is not sensitive to processes that are related to, but distinct from, WM, such as spatial attention or task difficulty (Feldmann-Wüstefeld et al., 2018; Ikkai et al., 2010; McCollough et al., 2007; Vogel & Machizawa, 2004; and for a review, see Luria et al., 2016). Similarly, the resetting-drop in CDA amplitude is extremely specific: When the mapping between WM representations and perceptual input is disrupted there is a characteristic drop, whereas extremely similar situations (even within experiment and participants) that allow this mapping to hold lead to a smooth change in amplitude (Balaban et al., 2018a, 2019; Balaban & Luria, 2017; and for a review, see Balaban & Luria, 2019). Last though crucial, the CDA-drop does not reflect general surprise. Rather, it is the result of a specific disruption to object tracking. The effect persists after many dozens of exposures to the disrupting events, and can also be observed for events that are completely predictable (Balaban et al., 2023).

Based on prior work, we analyzed the CDA in several predefined time-windows (for a similar approach in different contexts, see Balaban & Luria, 2015, 2016a, 2016b; Drew et al., 2012, 2013; Luria & Vogel, 2011a; Peterson et al., 2015). First, to examine tracking, for each condition we compared mean amplitude across two previously-defined time-windows (Balaban & Luria, 2017): the resetting window, 200–300 ms after the critical event, and the pre-resetting baseline window, immediately preceding it, i.e., 100–200 ms after the critical event. Here, the critical event was the moment when items started to emerge from behind the occluder, or, in the Vanish condition, the time in which an item should have emerge but didn’t. This happened 1896 ms after the onset of the video. Note that this moment refers to the first frame in which objects can potentially be seen behind the occluder (which is after the moment when the occluder starts to lower, but before it is fully dropped). Because this is a perceptually non-salient change in the visual input, time-locking in our study might be less precise than in previous related work. This in turn could cause the resetting effect to be more smeared in time. We return to this point in the Discussion.

We additionally examined the window immediately following the resetting window (300–400 ms after the critical event, compared to the pre-resetting baseline). In previous studies involving object separation, this delay was enough time for the resetting process to finish and the amplitude to recover (e.g., Balaban & Luria, 2017).

To establish scene reinterpretation, we compared mean amplitude during the retention interval (3200–3900 ms after trial onset; note that the CDA takes about 200 ms to respond, e.g., to initially rise, see Vogel et al., 2005) across the different conditions. Specifically, we examined whether, after the video ended, participants represented the Create and Vanish conditions similarly to 1 object or to 2 objects (i.e., whether the amplitude in each impossible condition is lower than that of the 2-Objects Control, or higher than that of 1-Object Control). Additionally, in the Supplemental we report the results of an exploratory analysis of the pre-occlusion period.

A resetting-drop was established via a within-subjects Analysis of Variance (ANOVA), with Time (pre-resetting baseline vs. resetting, and pre-resetting vs. prolonged resetting) and Condition as independent factors on mean amplitude as the dependent measure. The final representation was examined with a within-subjects ANOVA, with Condition as an independent factor, on mean amplitude during the retention time-window as the dependent measure. Finally, we analyzed behavioral performance in the task with a one-way within-subjects ANOVA with condition as an independent factor on accuracy as a dependent measure.

We followed these ANOVAs with planned comparisons (contrasts), the results of which we focus on, for simplicity. We additionally report effect sizes for all statistical comparisons, and 95% confidence intervals (CIs) for the difference between conditions. Despite strong predictions for the direction of effects, all tests were two-tailed to avoid potential biases.

## RESULTS

### Experiment 1: Active Representations Rely on Intuitive Physics

In Experiment 1, we tested how object permanence violations affect online maintenance and tracking. Within a WM task, participants (*N* = 16) watched animations of objects moving across a stage, behind a rising screen, and back out as the screen lowers (Figure 1A). In the Control conditions, the animations proceeded as expected (Videos S1 and S2). In the impossible conditions, while the screen was up an object was added (Create) or removed (Vanish), seeming to appear or disappear magically (Videos S3 and S4). Current views of object tracking suggest it should be sensitive only to low-level visual properties, rather than physical expectations. If this was true, the CDA should rise or fall smoothly without a resetting-drop, because the events we tested were either not salient because they occurred in an occluded area so perceptually were comprised only of something that should have happened but didn’t (Vanish), or included an object that wasn’t previously tracked (Create). However, if events that contradict intuitive physics disrupt the ongoing function of the pointer system (as we hypothesize), then both Create and Vanish should trigger a CDA-drop (resetting).

**Figure 4.**
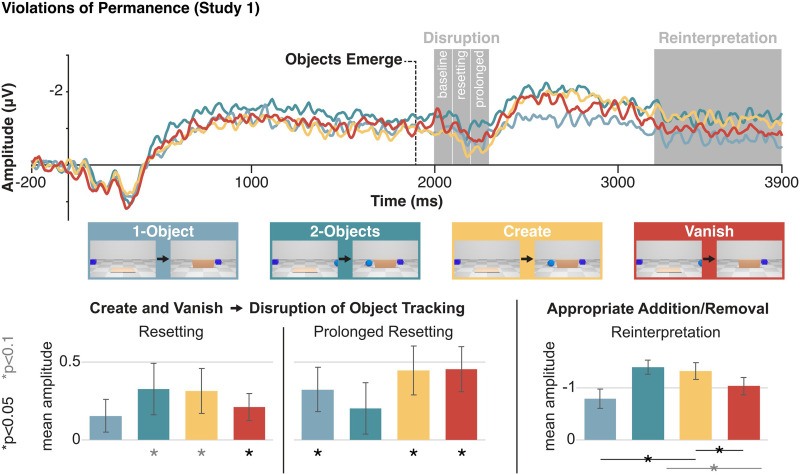
EEG results for Experiment 1. Top: CDA waveforms by condition. Dashed line indicates when objects emerge behind the lowering screen. Analyzed time-windows are in grey. Bottom: Mean amplitude by condition and time-window; error bars show standard error. From left to right: Resetting minus baseline (indicating object tracking disruption), immediately following window minus baseline (indicating prolonged object tracking disruption), and retention interval amplitude (indicating scene reinterpretation, i.e., the number of represented objects at the end of the trial). Asterisks show significant (black) and marginally significant (grey) contrasts, following the ANOVAs (see Methods).

To test the possible disruption of the pointer system, we compared the CDA amplitude in the resetting time-window (200–300 ms after the critical event, when an item appeared or failed to appear) to the baseline time-window which immediately precedes any potential CDA-drop (Balaban & Luria, 2017). We also compared the baseline to the immediately subsequent time-window (300–400 ms after the critical event). After establishing a significant interaction of Time and Condition (*F*(3, 45) = 3.31, *p* = 0.028), we focused our analysis on the violation conditions. We found a CDA-drop for both types of object permanence violation (Create and Vanish), with lower amplitude in the resetting time-window (Figure 4). This effect was marginally significant in the Create condition (*F*(1, 15) = 4.47, *p* = 0.05, *d* = 0.5, 95% CI = [0.0, 0.63] *μ*V), and significant in the Vanish condition (*F*(1, 15) = 5.61, *p* = 0.03, *d* = 0.6, 95% CI = [0.02, 0.4]). As detailed above, this CDA-drop does not reflect a general surprise from some event, but specifically marks a disruption to the ongoing function of the pointer system (the effect can be found after numerous exposures to a specific event, and even when an event is perfectly predictable). So, the results demonstrate that the pointer system depends on intuitive physics for object tracking.

Resetting even persisted in the following time-window, with significantly lower amplitudes (as compared with the pre-resetting window) for both the Create (*F*(1, 15) = 7.67, *p* = 0.01, *d* = 0.7, 95% CI = [0.1, 0.79]) and Vanish (*F*(1, 15) = 9.08, *p* = 0.01, *d* = 0.8, 95% CI = [0.13, 0.78]) conditions. In previous studies that used 2D events like separation, this delay was enough for the system to recover (Balaban et al., 2019), and so the prolonged resetting effect in the current setup might reflect the fact that the pointer system took longer to recover from violations of object permanence. However, we cannot draw strong conclusions regarding recovery, for several reasons. First, we cannot directly compare the current results with experiments that use different stimuli. Second, our events were likely less temporally precise than previously used events, given the gradual and non-salient change in the scene as objects come out from behind the occluder or fail to do so. Third, recent evidence suggests that a CDA-drop in this later time window might reflect a specific sub-process of resetting, namely an interference between old and new representations, or a challenge in reforming a correspondence (Friedman et al., 2024), which fits the current violations of object permanence. So, the precise temporal profile of the influence of physical violations on online processing, and its implications for better characterizing the resetting process, remain open questions for future research.

In addition to the predicted resetting effects in the violation conditions, we found possible marginal evidence for shorter-lived resetting effects in the Control conditions. In the resetting time-window, there was borderline evidence for a drop in the 2-Objects Control (*F*(1, 15) = 3.66, *p* = 0.08, *d* = 0.5, 95% CI = [−0.04, 0.69]), and no evidence for this effect in the 1-Object Control (*F*(1, 15) = 1.97, *p* = 0.18, *d* = 0.4, 95% CI = [−0.08, 0.39]). In the later time-window, there was no evidence for resetting in the 2-Objects Control (*F*(1, 15) = 1.38, *p* = 0.26, *d* = 0.3, 95% CI = [−0.17, 0.57]), and there was an effect for the 1-Object Control (*F*(1, 15) = 4.88, *p* = 0.04, *d* = 0.6, 95% CI = [−0.01, 0.64]). We believe these effects, if they can be considered that, are artifacts. Beyond the fact that the evidence for them was not strong in this experiment, the results of Experiment 2 (see below) show that a direct replication of the Control conditions result in no effect at all. Still, if one were to try to account for these possible effects, we would suggest that the violations of intuitive physics in the impossible conditions may have led participants to undergo a resetting process in all conditions, though more weakly in the Control conditions. In support for this possibility, it has recently been shown that when pointer-disruption events become prevalent, a resetting process will occur in situations that do allow the mapping to hold (Friedman et al., 2024). See also the Supplemental for suggestive support for the possibility that this might be a strategic shift. Importantly, a resetting effect in the control conditions does not go against the theory that physical expectations influence the pointer system, because without this influence the expected pattern would be to find no resetting in *any* condition.

In addition to tracking disruption, we compared the final CDA amplitude (during the retention interval) of the different conditions, to test how WM representations changed as the scene evolved. We found a significant effect of Condition (*F*(3, 45) = 4.46, *p* = 0.008). We expected that by the end of the trial, the CDA amplitude would reflect the updated number of items in the scene. In line with this, a comparison of the different conditions indicated that the CDA amplitude was consistent with an item being added in the Create condition, and removed in the Vanish condition. The CDA amplitude in Create was higher than the 1-Object Control (*F*(1, 15) = 9.77, *p* = 0.01, *d* = 0.8, 95% CI = [0.17, 0.9]), and similar to the 2-Objects Control (*F* < 1, *p* = 0.76, *d* = 0.1, 95% CI = [−0.41, 0.55]). The CDA amplitude in the Vanish condition was similar to the 1-Object Control (*F*(1, 15) = 1.81, *p* = 0.2, *d* = 0.3, 95% CI = [−0.13, 0.57]), and lower than the 2-Objects Control, though this comparison was only marginally significant (*F*(1, 15) = 3.94, *p* = 0.07, *d* = 0.5, 95% CI = [−0.03, 0.8]). Importantly, the Create condition had a higher amplitude than the Vanish condition (*F*(1, 15) = 6.36, *p* = 0.02, *d* = 0.6, 95% CI = [0.05, 0.58]). These findings indicate that participants successfully and rapidly adjusted their representations after object permanence violations, adequately adding or removing objects from WM.

For completeness, we also examined behavioral performance, and found a significant effect of Condition on accuracy (*F*(3, 45) = 41.69, *p* < 0.001). We stress that accuracy in our task does not tap into the ongoing dynamics of representations in WM, given the long presentation time after items exit from behind the occluder. The mean accuracy in the Create condition was lower than in the 1-Object Control (*M* = 0.78 vs. 0.93; *F*(1, 15) = 100.8, *p* < 0.001, *d* = 2.5, 95% CI = [0.12, 0.19]), and similar to the mean accuracy in the 2-Objects Control (*M* = 0.79; *F* < 1, *p* = 0.4, *d* = 0.2, 95% CI = [−0.02, 0.04]). These results match the item load at the end of the trial. The mean accuracy in the Vanish condition was lower than in the 1-Object Control (*M* = 0.88; *F*(1, 15) = 31.1, *p* < 0.001, *d* = 1.4, 95% CI = [0.03, 0.08]), and higher than the 2-Objects Control (*F*(1, 15) = 20.8, *p* < 0.001, *d* = 1.1, 95% CI = [0.05, 0.13]) and Create (*F*(1, 15) = 68.4, *p* < 0.001, *d* = 2.1, 95% CI = [0.08, 0.13]) conditions. This pattern of results suggests that behavioral performance in our task mainly reflects a combination of two factors: the number of items one holds in WM (which can be assessed by the CDA amplitude at the end of the trial), and the amount of encoding resources participants could allocate to each item. This later factor is likely heavily influenced by the relative time available to encode each item. So, in the Vanish condition participants eventually represented only a single object, but this object shared half of its presentation time with another object, thereby decreasing the effective encoding resources and accordingly, accuracy, relative to the 1-Object Control. Why then is accuracy in the Create condition similar to the 2-Objects Control? We hypothesize that this reflects two opposite effects that cancel each other out. Relative to the two items in the Control condition, the originally presented item in the Create condition received more encoding resources (because it appeared on its own for a relatively long time), while the newly added item received less encoding resources (because it was presented for less time). Corroborating this speculation, accuracy in the Create condition when the first item was probed at test (*M* = 0.83) was higher than when the second item was probed (*M* = 0.73; *t*(15) = 3.3, *p* = 0.005, *d* = 0.8, 95% CI = [0.24, 1.38]).

### Experiment 2: Online Representations Depend on Expectations

In Experiment 2, we tested whether changing the interpretation of scenes can affect the moment-by-moment dynamics of object maintenance and tracking. New participants (*N* = 16) watched short animations that were identical to Experiment 1, except that the Create and Vanish conditions included a small black rectangle behind the rising screen (Figure 1B; Videos S5 and S6), which was described to participants as a hole that objects could climb up from or fall into. The hole was hidden behind the screen when objects emerged, making the scenes perceptually-identical to Experiment 1 during the critical moments. The two experiments diverged only in terms of participant expectations, as the appearance or disappearance of items could now be explained away (Perez & Feigenson, 2022). So, if perceptual object tracking and WM representations are not directly influenced by physical expectations, we should expect the same pattern of results as in Experiment 1. However, if physically explaining away the violations via the presence of a hole interacts with processing, we would expect a different pattern of results. Most critically, we would expect that the resetting effect should diminish (at least for the Vanish condition, where the event is in line with gravity).

**Figure 5.**
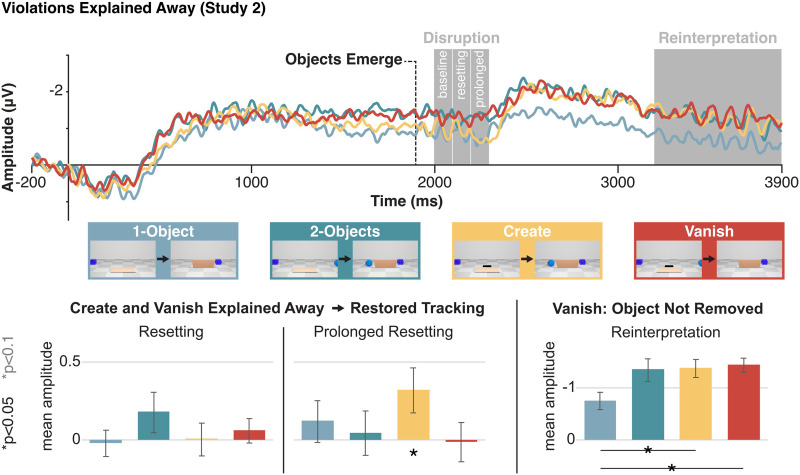
EEG results for Experiment 2. Top: CDA waveforms by condition. Dashed line indicates when objects emerge behind the lowering screen. Analyzed time-windows are in grey. Bottom: Mean amplitude by condition and time-window; error bars show standard error. From left to right: Resetting minus baseline (indicating object tracking disruption), immediately following window minus baseline (indicating prolonged object tracking disruption), and retention interval amplitude (indicating scene reinterpretation, i.e., the number of represented objects at the end of the trial). Asterisks show significant (black) and marginally significant (grey) contrasts.

As in the previous experiment, we examined potential disruptions to the pointer system by comparing the resetting time-window, and the subsequent time-window, to the baseline time-window immediately before any CDA-drop, and found a significant interaction of Time and Condition (*F*(3, 45) = 4.18, *p* = 0.01). If physical expectations govern online object tracking over and above low-level factors, then making the Create and Vanish scenes physically possible should eliminate the resetting effect. Accordingly, as can be seen in Figure 5, we found that there was no resetting effect for Create nor Vanish (both *F*’s < 1, both *p*’s > 0.4, both *d*’s < 0.2; Create 95% CI = [−0.22, 0.23]; Vanish 95% CI = [−0.11, 0.23]). In the following time-window, there was no resetting effect for Vanish (*F* < 1, *p* = 0.9, *d* = 0, 95% CI = [−0.26, 0.28]), but there was one for Create (*F*(1, 15) = 4.59, *p* = 0.049, *d* = 0.5, 95% CI = [0.0, 0.63]). This resetting effect in the Create condition is likely due to the fact it was more difficult to explain an appearance (because the new object must go against gravity to climb up from the hole), and to predict which exact object will emerge. As mentioned above, in the present setup, the interpretation of a resetting effect in the later time-window is ambiguous: It might reflect a ‘classic’ resetting effect that is more temporally-smeared due to the nature of the realistic stimuli used in the present study, or a difficulty in reestablishing a correspondence between the perceptual input and WM’s representations, due to interference between the original and new items (see Friedman et al., 2024).

We next examined whether there was a resetting effect in the Control conditions, which were identical to the Control conditions of Experiment 1. As a reminder, we found some weak evidence for this effect in the Control conditions of Experiment 1. In Experiment 2, we found no drop effect in the Control conditions, in either the resetting time-window (1-Object Control: *F*(1, 15) = 1.76, *p* = 0.2, *d* = 0.3, 95% CI = [−0.11, 0.45]; 2-Objects Control: *F* < 1, *p* = 0.8, *d* = 0.1, 95% CI = [−0.16, 0.2]) or the prolonged resetting window (both *F*’s < 1, both *p*’s > 0.3, both *d*’s < 0.3; 1-Object Control 95% CI = [−0.17, 0.41]; 2-Objects Control 95% CI = [−0.26, 0.35]). The lack of a drop effect shows that a resetting process isn’t mandatory across all occlusion events. Because the Control conditions were identical in Experiment 1 and 2, the non-effect in Experiment 2 suggests that the Control events alone are not sufficient to trigger a resetting process, which is in line with the idea that in Experiment 1, any drop in the Control conditions is likely due to the context produced by the impossible conditions, as suggested in recent work (Friedman et al., 2024).

Our main analyses focus on comparisons of conditions or time-windows within each experiment. But as an additional post-hoc analysis, we compared the resetting effects across the two experiments. The three-way interaction of Experiment, Condition, and Time was marginally significant (*F*(3, 90) = 2.37, *p* = 0.076). The effects in the control conditions did not differ across experiments (all *t*’s < 1, all *p*’s > 0.2). While interpreting null effects is problematic, especially considering the likely low power of the present comparison, this pattern fits our interpretation of the effect in Experiment 1’s control conditions as unreliable. In the Create condition, there was a non-significant trend towards a larger effect in Experiment 1 in the earlier time window (*t*(30) = 1.7, *p* = 0.09), and no difference in the later time window (*t* < 1). Conversely, in the Vanish condition, the resetting effect in Experiment 1 was larger than in Experiment 2, though this effect was only significant in the later time-window (*t*(30) = 2.35, *p* = 0.025) and not the earlier one (*t*(30) = 1.28, *p* = 0.2). We again stress that these between-subjects comparisons were not planned in advance and are likely under-powered, but the obtained pattern supports our interpretation of the results, such that the presence of the hole in Experiment 2 reduced the resetting effect.

As with Experiment 1, we established how the scenes are interpreted after all the events took place by comparing the final CDA amplitude across the different conditions, which resulted in a significant effect of Condition (*F*(3, 45) = 4.46, *p* = 0.008). We found that in the Create condition, the final CDA amplitude followed the updated number of objects in the scene, in that it was higher than the 1-Object Control (*F*(1, 15) = 11.4, *p* = 0.004, *d* = 0.8, 95% CI = [0.23, 1.02]), and similar to the 2-Objects Control (*F* < 1, *p* = 0.9, *d* = 0.0, 95% CI = [−0.41, 0.47]). Most interestingly, in the Vanish condition the amplitude was also significantly above the 1-Object Control (*F*(1, 15) = 11.57, *p* = 0.004, *d* = 0.9, 95% CI = [0.26, 1.13]), and similar to the 2-Objects Control (*F* < 1, *p* = 0.7, *d* = 0.1, 95% CI = [−0.4, 0.6]), as well as the Create condition (*F* < 1, *p* = 0.7, *d* = 0.1, 95% CI = [−0.4, 0.6]). This result suggests that participants continued to hold the vanished object in WM, even though, as in Experiment 1, the object disappeared from view, and was never probed during the memory test. The item that ‘fell down the hole’ is out of view, but still part of the scene. This suggests that online representations are not determined only by visual factors, or simply by predictability (which would lead participants to never represent the to-be-vanished item), but also by physical explanations.

Again for completeness, we examined people’s behavioral performance on the memory task, finding a significant effect of Condition on accuracy (*F*(3, 45) = 33.89, *p* < 0.001). We found the same pattern as in Experiment 1, corroborating the claim that the physical violations in our study were task-irrelevant. The mean accuracy in the Create condition was lower than in the 1-Object Control (*M* = 0.79 vs. 0.93; *F*(1, 15) = 61.47, *p* < 0.001, *d* = 2.0, 95% CI = [0.09, 0.16]), and similar to the 2-Objects Control (*M* = 0.8; *F*(1, 15) = 1.71, *p* = 0.2, *d* = 0.3, 95% CI = [−0.01, 0.04]). The mean accuracy in the Vanish condition was lower than in the 1-Object Control (*M* = 0.88; *F*(1, 15) = 11.58, *p* = 0.004, *d* = 0.9, 95% CI = [0.02, 0.09]), and higher than the 2-Objects Control (*F*(1, 15) = 16.52, *p* = 0.001, *d* = 1.0, 95% CI = [0.03, 0.1]) and Create (*F*(1, 15) = 21.92, *p* < 0.001, *d* = 1.2, 95% CI = [0.05, 0.12]) conditions. So, while people’s online representational dynamics differed across Experiments 1 and 2, their behavioral memory performance was the same. The general pattern is in line with our suggestion that accuracy is mainly driven by the number of items held and the encoding resources they received, including a replication of Experiment 1’s finding of better accuracy in the Create condition when the first item was probed (*M* = 0.82) than when the second item was probed (*M* = 0.76; *t*(15) = 4.2, *p* < 0.001, *d* = 1.1, 95% CI = [0.43, 1.6]). But, given that the CDA indicated that participants kept on representing both items in the Vanish condition (despite knowing they won’t be probed on the item that disappeared), why was performance not as low as for two items? This might reflect a cueing effect. It is well documented in WM tasks that when several items are presented during a memory array and one of them is cued (either during encoding, or even later as in a ‘retro-cue’), performance is better than without such a cue (e.g., Griffin & Nobre, 2003; Landman et al., 2003; Vogel et al., 2005). In a similar way, the Vanish condition might have allowed participants to prepare well in advance for the memory test, because they knew which item will be probed.

## DISCUSSION

Our findings show for the first time how violations of intuitive physics are processed moment-by-moment to support object tracking and WM updating. We used scenes inspired by developmental studies and AI benchmarks, but presented them in a WM task and employed an approach previously unused in this context, which allowed us to uncover the hidden neural dynamics of processing physically surprising events that are irrelevant to the task. Specifically, we examined the CDA (Luria et al., 2016; McCollough et al., 2007; Vogel & Machizawa, 2004), an ERP index of online processing, in two different ways, one tackling the representational contents of WM, and the other focusing on the mapping between these representations and ongoing perception, which is carried out by the pointer system that tracks objects. Because pointers are theorized to not carry the representational content themselves (Pylyshyn, 2000, 2004), we explicitly treat these two aspects as distinct (note that some recent conceptions are somewhat different, e.g., Thyer et al., 2022). It was also recently demonstrated that pointers can be experimentally dissociated from representations, such that two objects are compressed into a single representations while maintaining two independent pointers (Balaban et al., 2023). As our first measure, the presence or absence of a CDA-drop after a given event indicated whether this event prevented objects in the scene from being continuously tracked, causing a resetting process of removing the original representations and starting anew (Balaban et al., 2018a, 2019; Balaban & Luria, 2017; for behavioral evidence that representations are inaccessible during resetting, see also Balaban et al., 2018b). Second, comparing the CDA amplitude between different conditions showed how the scenes were represented in WM after each event took place.

The novel use of a well-established electrophysiological marker revealed that violations of object permanence disrupt the pointer system’s ability to track objects (Experiment 1), due to expectations about physical outcomes (Experiment 2). The disruption of tracking during violations of physical expectations sheds light on the principles governing the normal function of the adult pointer system. Most importantly, in maintaining a continuous correspondence between the perceptual input and the active representations in WM, the pointer system is not solely driven by low-level factors, as is usually assumed in discussions of object tracking (for a review of this subject, see Holcombe, 2023). While the pointer system obviously relies on visual input, the present results show that it also incorporates the physical interpretation of visual events (Lau & Brady, 2020).

But the results go further, in specifying several aspects of the relationship between tracking and intuitive physics. First, the presence of a resetting effect in the Vanish condition shows that an event does not have to be perceptually salient to disrupt object tracking. Second, the resetting effect in the Create condition suggests that the pointer system is sensitive to the physical aspects of the entire scene in tracking objects: The new object’s appearance is only “impossible” in the sense that it should not have appeared in a previously-empty place (notably, the mere appearance of a new object in a scene does not in itself trigger resetting; Balaban & Luria, 2017). Third, in Experiment 2 a disappearing object did not disrupt tracking once the event became physically possible, despite being perceptually-identical to Experiment 1 during the critical time-windows, suggesting the pointer system relies not only on visual information but on physical explanations.

Our findings suggest that the brain’s tracking system predicts an object’s future location based on intuitive physics, perhaps via noisy quantitative physical simulation (Battaglia et al., 2013; Smith et al., 2019; Ullman et al., 2017). Our results help explain numerous separate past findings, which showed pointer system resetting following violations of object separation (Balaban et al., 2018a, 2018b, 2019; Balaban & Luria, 2017), object replacement (Balaban & Luria, 2017; Friedman et al., 2024), and feature switching (Park et al., 2020). While these situations were not originally explained in such a way, they violate cohesion, object permanence, and kind-identity, respectively. Importantly, these past effects included salient changes to the tracked object itself, while here we found that even violations that are not directly observed (Vanish) or involve a novel object (Create) disrupt tracking, while *visually-identical* displays with a different physical interpretation do not disrupt tracking (Vanish condition of Experiment 2). This specifically marks intuitive physical expectations as directly shaping the pointer system’s tracking ability, in a way that is not mediated by visual factors.

The CDA further allowed us to decipher people’s flexible interpretation of events, and how it changes to fit the inferred meaning of the unfolding scene: WM recovers after resetting within the time frame examined, and scene representations are correctly adjusted to add or remove objects (Experiment 1), based not only on what is available in perception, but also on the physical explanations of events (Experiment 2). Specifically, the different final representation of the Vanish condition in each experiment (which was similar to a single object after disappearing in a physically-impossible way in Experiment 1, but similar to two objects after disappearing in the physically-possible, visually-identical, way in Experiment 2) highlights how common-sense physical understanding shapes WM’s online *contents*—over and above low-level visual properties.

Importantly, while the current setup relates to several previous behavioral studies focusing on the importance of spatiotemporal information in WM representations and online tracking (e.g., Flombaum & Scholl, 2006; Flombaum et al., 2009; Mitroff et al., 2004; Schurgin & Flombaum, 2017), it goes beyond them in two key ways. First, due to the nature of the ERP technique and the specific components we isolated, we could pin-down the effects to particular substages of online processing, namely to dissociate influences relevant to the pointer system’s tracking capacity from those relevant to the representational content of WM. Second, the present findings cannot be reduced to violations of spatiotemporal continuity, which previous research highlighted as central to both tracking and object representations. This is because across the two experiments, each condition was fixed in terms of spatiotemporal cues, and so the fact that the results differed between them—both in terms of the pointer system’s ability to track objects (as indexed by the presence or absence of a CDA-drop) and in terms of WM’s representational contents (as indexed by the final CDA amplitude)—shows that spatiotemporal information alone cannot be the sole driving force here. While we definitely accept its importance, we argue that spatiotemporal continuity should be considered as one key aspect of intuitive physical knowledge, which is the factor that the current study suggests determines online processing.

The results of our experiments do not reflect surprise itself (in the sense of ‘statistical’ surprise by a rare event, or saliency-driven ‘perceptual’ surprise), but rather a disruption of the pointer system. We found this system to be sensitive to physical violations, which could feed downstream to a surprise signal. The experiments also show that pointer system disruption can be mitigated by expectations, but that this mitigation depends on the format of the expectations. In Experiment 1, participants saw the stimuli dozens of times, which forms the overall statistical expectation that in principle objects can sometimes disappear or appear in the videos. This statistical expectation did not prevent a robust and repeated tracking disruption. Conversely, the expectations in Experiment 2 were successful in eliminating the disruption. But, these expectations had a perceptual and causal format tied to a specific stimulus: the hole in the ground explained causally why on this trial an object might disappear or appear, and did so in the same visual format as the rest of the stimuli. Past studies of resetting in the pointer system support the distinction between expectation formats: numerous exposures to a disruptive event did not eliminate the disruption, and even making a disruptive event perfectly predictable failed to do so (Balaban et al., 2023), but subtle visual cues that altered the way the scene was carved into distinct objects, thereby changing the targets for tracking, were successful right away (e.g., Balaban et al., 2019). Future work can adopt the present approach to further examine how the pointer system communicates with other processes, by looking at what other physical situations and expectations disrupt or preserve object tracking, including perceptually weak but causally specific expectations such as telling participants the rising screen acts as a magic box on specific trials. While more work is needed, our conclusion is not just that the pointer system’s ability to track objects is based on physical reasoning, but that the format of this physical reasoning matters.

Along similar lines, an interesting open question concerns the conditions necessary for WM to overcome the resetting process triggered by physical violations. We found that hundreds of repetitions of the violating events over the course of an hour or two were not enough to negate the effect. Note that after so many exposures, people might even report being bored by these stimuli (they are no longer overtly ‘surprised’), but they still consider the events to be impossible. We see several options regarding the possibility of making a violating effect no longer impossible. It is conceivable that intuitive physical expectations are too deeply rooted to overcome regardless of evidence to the contrary. Alternatively, significant exposure (perhaps over several days) could be enough to wash away the very heavy prior given to physical core knowledge, or maybe that stable enough exposure to violations (instead of mixing these events with possible ones) would do, or that the exposure must involve ‘real world’ violations instead of animations. Finally, another intriguing possibility is that exposure alone will never do, and some top-down explanation must come along with it to be successful. Empirically teasing apart these alternatives in future research could reveal how flexible the tracking system is with regards to intuitive physics, and how penetrable core physical knowledge is.

Our findings introduce a novel way of tackling both new and outstanding open questions. Notably, when combining naturalistic videos with ERPs it is important to take into account that events might be much less temporally-precise than the highly stylized, perceptually-salient stimuli often used in visual cognition research. The temporal imprecision of natural events likely influenced our findings here, for example in how temporally smeared the resetting effect is. Striking the right balance between ecological validity and methodological rigor should be a major focus of this research going forward. The moment-by-moment analysis of online processing, tracking, and representations allows researchers to address the different sub-stages of intuitive physics that underlie overt surprise, and can tease apart generic surprise from the violation of intuitive physics more specifically. For example, a mug falling off a table and bouncing, which could reveal that it is made out of rubber, is a physically surprising event that we hypothesize might not interfere with tracking, because it involves a single, uninterrupted object. In the psychological domain, both adults and young children are surprised when agents act in an inefficient way (e.g., Baillargeon et al., 2016). Nevertheless, we would predict such surprise would not lead to resetting, as this violation does not interfere with object tracking.

Our results show that scene representations in WM are adjusted following impossible events. The details of how people generate specific interpretations of physical scenes, and their direct correspondence to tracking remains to be tested. Future studies can leverage the CDA, individual patterns and time-courses of representations to examine correspondence to people’s reported explanations. Ideally such experiments would involve ambiguous scenes with multiple possible explanations that correspond to different numbers of objects to be maintained. As just one such example, consider stimuli used to test solidity in infants, such as a screen rotating in a way that first occludes, then seem to ‘move through’ a solid body such as a teddy bear or ball (Baillargeon, 2004). When adults see such stimuli, most explain it as the object having unexpectedly ‘disappeared’, while a minority explain it as the object being unexpectedly elastic (Smith et al., 2020). Such different explanations would correspond to different patterns of CDA amplitude and resetting.

A related and exciting avenue for future research involves constructing a developmentally appropriate version of measuring a CDA-like pattern in infants. Such an approach could potentially tease apart distinct underlying sources of overt surprise signals such as looking time. For example, it might establish that the overt surprise shown by infants in response to violations of physical expectations sometimes reflects disrupted physics-based object tracking, while in other cases it is not rooted in the disruption of tracking.

More generally, our method can shed light on how people perceive, track, understand, and remember physical events, by bridging traditional cognitive science, developmental psychology, and neuroscience perspectives. We see great promise in hybrid approaches that, as we do here, use simplified but ecologically foundational stimuli based on infant studies, while measuring well-characterized neural markers resting on years of rigorous quantitative validation from carefully controlled psychological experiments with adults. Examining the CDA in this novel manner allowed us to reveal previously-hidden influences of intuitive physics on scene representations and object tracking.

## ACKNOWLEDGMENTS

We thank Roy Luria for his generosity in providing the experimental site.

## FUNDING INFORMATION

This work was supported by the Defense Advanced Research Projects Agency (DARPA) Machine Common Sense program, the Center for Brain, Minds, and Machines (CBMM), Israel Science Foundation (ISF) Grant No. 2067/24 (HB), the Alon scholarship (HB), National Science Foundation (NSF) Grant No. 2121009 (KAS, JBT), NSF Grant No. 2124136 (JBT), and the Jacobs Foundation (TDU).

## AUTHOR CONTRIBUTIONS

HB: Conceptualization, Data curation, Formal analysis, Writing – original draft, Writing – review & editing. KAS: Conceptualization, Writing – original draft, Writing – review & editing. TDU: Conceptualization, Supervision, Writing – original draft, Writing – review & editing. JBT: Conceptualization, Supervision, Writing – review & editing.

## DATA AVAILABILITY STATEMENT

Data, code, and video examples are available at the Open Science Framework: https://osf.io/csarg/.

## Supplementary Material














