# Supplementary material for: Electrophysiology Reveals That Intuitive Physics Guides Visual Tracking and Working Memory
Source: Open Mind (Camb). 2024 Nov 22;8:1425–46. doi: 10.1162/opmi_a_00174 (PMC11634321; doi:10.1162/opmi_a_00174)
Supplement: Supplementary file 1 [file opmi-08-1425-s001.pdf]

Supplemental Information for
Electrophysiology reveals that intuitive physics guides visual tracking and working memory

Pre-Violation Analysis

Our task was not designed to test items' representations before they enter the occluder: While we did include catch trials to ensure participants were generally attending the scene, these 300-ms trials ended long before objects entered the occluder, not alone came out, so if participants saw that the trial did not terminate early they could suspend their detailed encoding of objects' features until objects exit from behind the occluder. In support for this, an exploratory analysis of accuracy in the catch trials showed that participants were much worse than in full-length trials (though still well above chance, which is at a level of 25% in our task), with an average of 0.59 for catch trials with two objects, and 0.85 for one.

We nevertheless examined, as a post-hoc and accordingly careful source of insight, the amplitude in an occluded time-window (1400-1800 ms from trial onset), comparing the amplitude across the different conditions in a similar manner to the final CDA amplitude. In Experiment 1, we found a higher amplitude in the 2-Objects Control than in the 1-Object Control ($t(15) = 2.18$, $p = 0.046$, $d = 0.55$) or the Create condition ($t(15) = 3.05$, $p = 0.008$, $d = 0.76$). Amplitude in the Vanish condition was also higher than in the 1-Object Control, but not significantly ($t(15) = 1.3$, $p = 0.2$, $d = 0.3$). In Experiment 2 the 2-Objects Control was again higher than the 1-Object Control ($t(15) = 3.64$, $p = 0.002$, $d = 0.91$) and marginally higher than in the Create condition ($t(15) = 1.86$, $p = 0.08$, $d = 0.47$). The Vanish condition was now significantly higher than the 1-Object Control ($t(15) = 3.17$, $p = 0.006$, $d = 0.79$). We interpret this as showing that before the items exit from behind the occluder the correct number of objects is generally represented, but the waveforms are likely too noisy to draw strong conclusions regarding this time-window in the current setup.

Split-Half Analysis

To examine how the different effects change throughout the course of the experiment, we split each session in two, and compared the results separately for each 8 blocks long half. Specifically, we wanted to test whether there were changes in the resetting effect in the control conditions as evidence accumulate, and also test whether there were hints for a strategic shift in participants' encoding of the items in the pre-occlusions stage. We therefore focus our analysis on the 1-Object and 2-Objects Control conditions. This analysis was not planned apriori, and so is likely underpowered, which is why we treat it as exploratory and qualitative. The results for both experiments are presented in Figure S1.

As can be seen in Figure S1, in Experiment 1 the results suggest that at least for two objects, a resetting effect was largely missing in the first half of the experiment, and present only in the second half. Conversely, in Experiment 2 the resetting effect (or lack thereof) was stable throughout the experiment. This fits our interpretation of the small resetting effect found in the full-experiment Control conditions as reflecting a 'resetting mode' (see Friedman et al., 2024) where a resetting process emerges for events that do not invalidate the ongoing function of the pointer system, due to exposure to invalidating events.

Another interesting finding relates to the time before items enter the occluder. In Experiment 1, there is a clear set-size effect during the first half, but no difference between the conditions in the second half. Again, this pattern doesn't occur in Experiment 2, where a

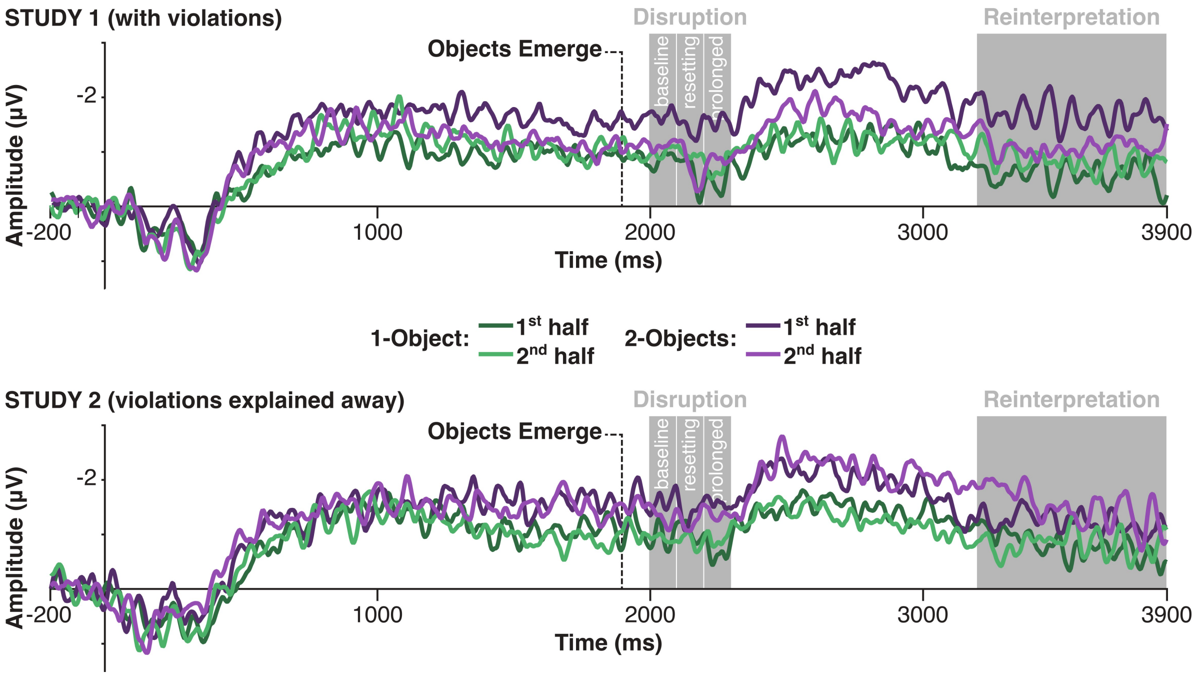

Figure S1: The EEG results in Experiment 1 (top) and 2 (bottom), by condition, split into the first and second halves of the task.

large set-size effect is present throughout the experiment. This suggests that in Experiment 1, participants gradually shifted to suspending their encoding of the objects' details until after they exit from behind the occluder, perhaps due to the possibility that a resetting process will be necessary.

Eye-Movement Analysis

Aside from removing trials with large eye-movements, to make sure there was no strong horizontal drift in any condition, we calculated the average horizontal EOG for left vs. right cue trials in the analyzed time-windows (Woodman and Luck, 2003). We found that eye-movements were small on average: the mean HEOG was 2.5 μV in Experiment 1, and 2.3 μV in Experiment 2, in Experiment 2, which translates to less than 0.2° of visual angle (Hillyard and Galambos, 1970). The HEOG across the different conditions ranged between 0.6 and 3.7 μV (translating to $0.04 - 0.24°$), with no significant difference across conditions. Figure S2 shows the residual HEOG activity across the different conditions.

Non-Lateralized Analysis

Our main analysis focuses on the CDA to interpret the way violations of physical expectations affect the abilities to track and represent information (via the resetting-drop and final amplitude, respectively). However, other ERP components might also provide insights into processes related to the reevaluation of information. Specifically, the P3 (or P300) compo-

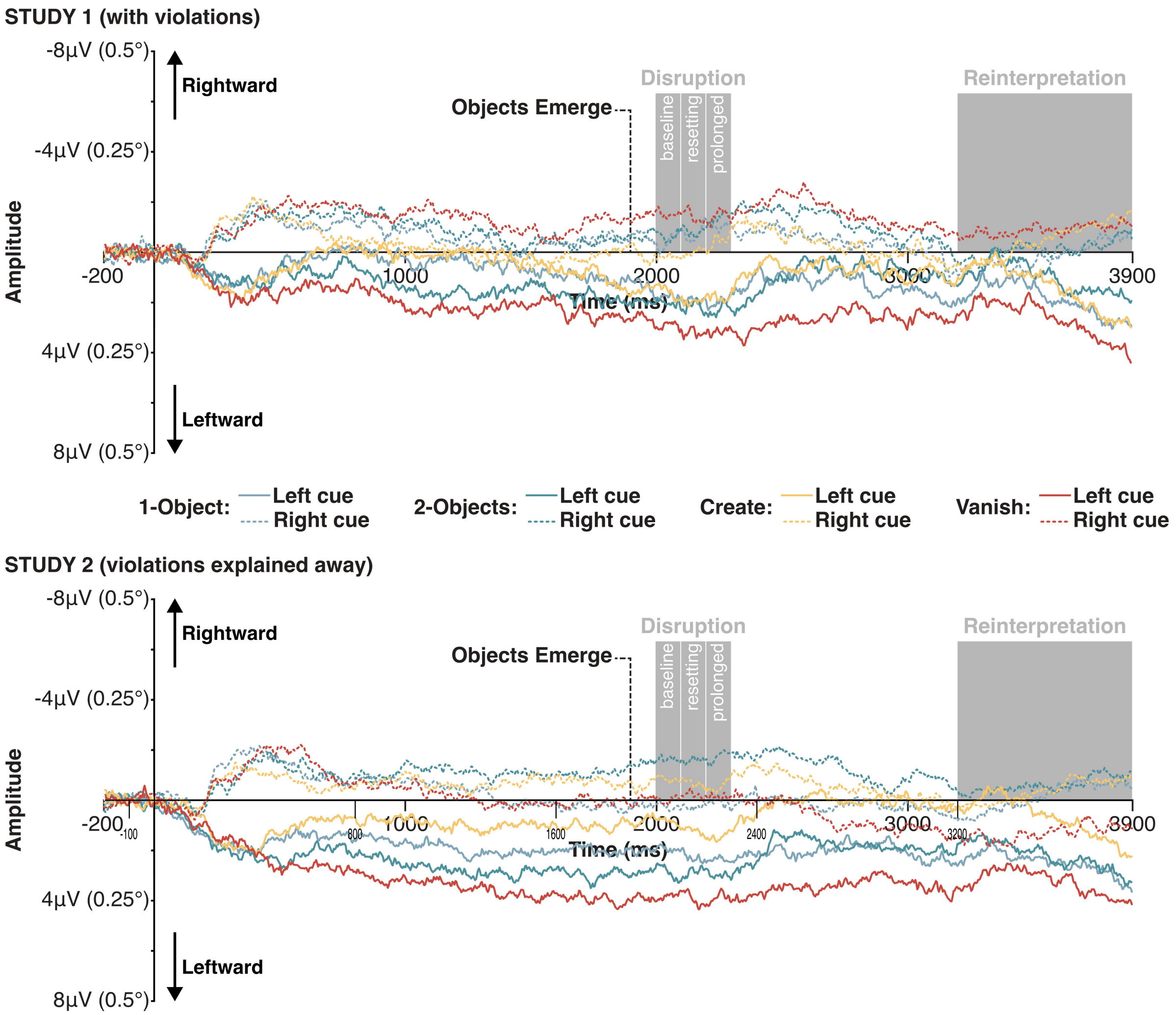

Figure S2: The HEOG activity in Experiment 1 (top) and 2 (bottom), by condition and attended side.

nent (for a review, see Polich, 2011) is often interpreted as reflecting context updating, and therefore it is interesting to examine how it is modulated by the current violation conditions. For completeness, we conducted a non-lateralized analysis and examined the mean amplitude across conditions, in the usual P300 electrodes, namely the Fz, Cz, and Pz electrodes. Notably, this analysis cannot subtract the ipsilateral from contralateral activity. The results are presented in Figure S3.

As can be seen in Figure S3, we do not get a classic P3 effect. While there is some positive deflection around the normal time-window of interest, i.e., 300-400 ms after the critical event, overall the waveforms are negative. Furthermore, the possible and impossible conditions are not neatly distinguishable, either within or between experiments. We refrain from interpreting the obtained pattern, and note that it would be very interesting to examine how physical violations are processed in a similar setup to ours but simply non-lateralized, so that a P3 can be clearly triggered.

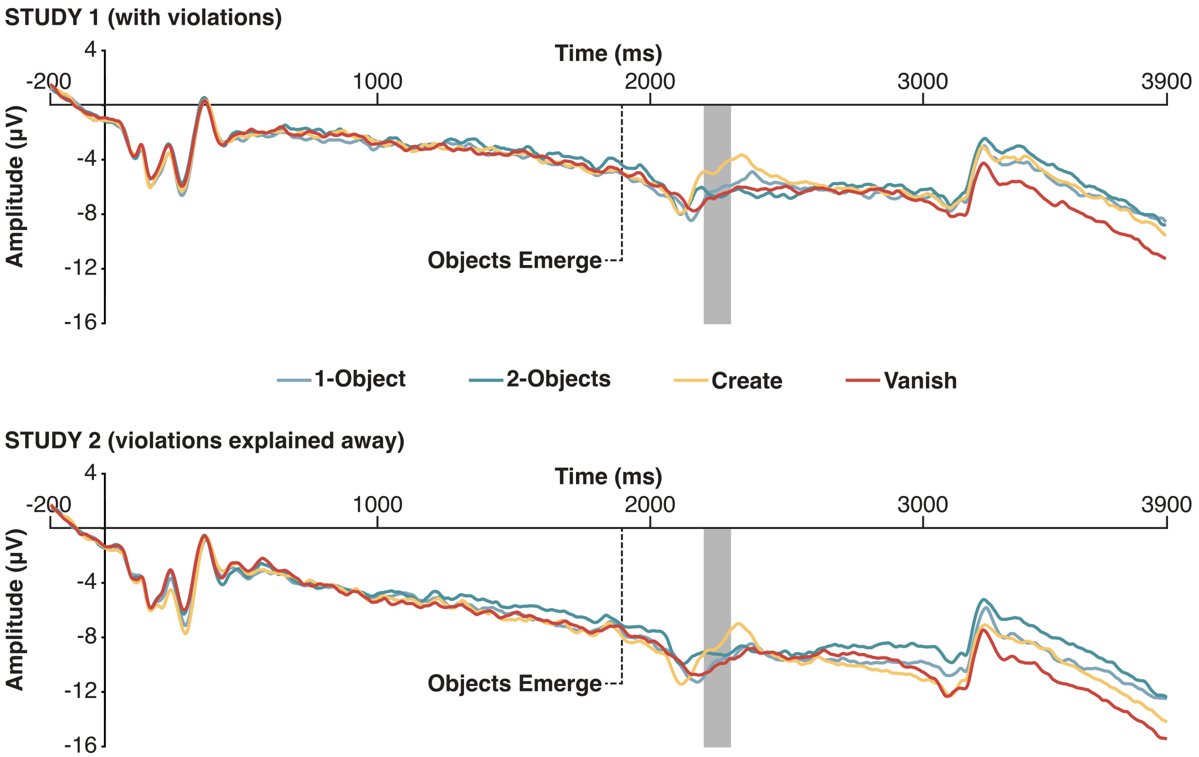

Figure S3: Average activity across the Fz, Cz, and Pz electrodes, by experiment and condition. Positive is plotted upwards, by convention in P3 research.

References

Friedman, S., Drew, T., and Luria, R. (2024). The effect of context on pointer allocation in visual working memory. *Cortex*, 177:170–179.

Hillyard, S. A. and Galambos, R. (1970). Eye movement artifact in the CNV. *Electroencephalography and Clinical Neurophysiology*, 28(2):173–182.

Polich, J. (2011). Neuropsychology of P300. In Kappenman, E. S. and Luck, S. J., editors, *The Oxford Handbook of Event-Related Potential Components*. Oxford University Press. _eprint: https://academic.oup.com/book/0/chapter/293241580/chapter-ag-pdf/44513063/book_34558_section_293241580.ag.pdf.

Woodman, G. F. and Luck, S. J. (2003). Serial deployment of attention during visual search. *Journal of Experimental Psychology: Human Perception and Performance*, 29(1):121–138.
